# Diagnosis and grading of adrenal cortical carcinoma

**DOI:** 10.1007/s00428-025-04278-0

**Published:** 2025-10-08

**Authors:** Giulia Vocino Trucco, Eleonora Duregon, Mauro Papotti, Marco Volante

**Affiliations:** 1 Pathology Unit, AOU San Luigi Gonzaga, Orbassano, Turin, Italy; 2 Department of Oncology, University of Turin, Città Della Salute E Della Scienza Hospital, Turin, Italy; 3 Department of Oncology, University of Turin, San Luigi Hospital, Regione Gonzole 10, Orbassano, Turin, 10043 Italy

**Keywords:** Adrenal cortical carcinoma, Diagnosis, Grading, Scoring, Classification

## Abstract

The 5th edition of the WHO classification of endocrine and neuroendocrine tumors represents a significant advancement in the diagnostic approach to adrenocortical carcinoma (ACC), integrating novel molecular insights with established histopathological criteria to enhance diagnostic accuracy and to refine prognostic assessment. This review outlines key histopathological features and diagnostic strategies for ACC, offering a practical framework for evaluation and grading in daily practice. The updated WHO classification reaffirms the central role of histopathology, employing multiparametric scoring systems that assess invasion, architectural and cytological features, mitotic activity, and necrosis. However, these parameters often pose interpretive challenges, and no single algorithm ensures complete sensitivity, specificity, or reproducibility. Therefore, combining diagnostic approaches is advisable, particularly in morphologically ambiguous cases. For tumor grading, the WHO employs a two-tiered system based on a mitotic count cut of 20 per 10 mm^2^, aiming to improve interinstitutional consistency. Immunohistochemistry remains essential for diagnostic confirmation and prognostic evaluation. Among available markers, SF1 is the most specific for adrenocortical origin, while Ki-67, mismatch repair proteins, p53, and β-catenin are useful for predicting patient outcomes or screening for hereditary predisposition. In this complex diagnostic setting, artificial intelligence holds potential to support ACC diagnostics. However, its application is limited by the rarity of the disease, histological variability, and the scarcity of large, well-annotated datasets necessary for algorithm development.

## Adrenal cortical carcinoma: a brief introduction

Adrenal cortical carcinoma (ACC) is a malignant tumor arising from adrenal cortical cells. It is a rare disease with an estimated incidence of 1 case per million in adults and 0.3 cases per million in children [1]. ACC is an aggressive disease with a dismal prognosis, accounting for the majority of deaths attributable to primary adrenal neoplasia [2] and with an estimated 5-year overall survival rate between 37 and 47% [3, 4].

ACC is usually a unilateral disease, with a preferential localization in the left adrenal gland [1, 5], whereas synchronous or metachronous bilateral involvement is rare. Exceptionally, ACCs may also occur in ectopic locations, and hitherto sporadic cases have been reported in the retroperitoneum, pelvic region, and ovary [6, 7].

Most ACC cases in adults occur sporadically. In this context, the most relevant etiologic factor is tobacco smoking [8, 9] with a two-fold greater incidence in smokers, which is even more pronounced in males.

Importantly, ACC is associated with a significant history of previous or subsequent associated cancers, thus suggesting heterogeneous underlying cancer predisposition mechanisms [9]. Associated malignancies are extremely variable, including different types of carcinomas, as well as testicular germ cell tumors, melanomas, lymphomas, and sarcomas. A proportion of ACC cases occur in the context of several germline susceptibility syndromes [10, 11]. Most commonly, these syndromic ACC cases were found in Li–Fraumeni syndrome, accounting for 3–5% of adult ACC cases [10, 11] and 50–80% of pediatric cases [10], but also Lynch syndrome [12, 13], Carney complex [14], familial adenomatous polyposis (FAP) [15], Beckwith–Wiedemann syndrome [16], multiple endocrine neoplasia type 1 (MEN1) [17], neurofibromatosis type 1 [18], and possibly subsets of the familial paraganglioma phaeochromocytoma syndromes [19], FH (hereditary leiomyomatosis and renal cell carcinoma syndrome) [20] or synchronous MSH2 and RET variants (without multiple endocrine neoplasia type 2) [21].

Most patients seek medical attention for symptoms related to hormone hypersecretion or for symptoms secondary to the compressive effects of an abdominal mass [22, 23]. However, with the increased adoption of advanced imaging techniques worldwide, a growing number of ACCs are now incidental findings and currently account for about 10% of cases [22].

## The WHO Classification (5th edition): a structural update

The 5th edition of the World Health Organization (WHO) classification of endocrine and neuroendocrine tumors introduced revisions to the diagnostic framework for ACC, integrating molecular insights with histopathological criteria to enhance diagnostic precision and prognostic relevance. These revisions aligned histological evaluation with contemporary molecular advancements in the fields of endocrine pathology, oncology, and molecular biology, offering a conceptual framework for tailored risk assessment and personalized management of ACC.

Importantly, the histopathological features remain the cornerstone of the diagnosis, and the main pathological characteristics as well as diagnostic tools are described in detail in this review.

Additionally, the WHO classification 5th edition emphasizes the importance of accurate proliferation metrics, such as mitotic counts and Ki-67 index. A significant update in the current classification is the shift from reporting mitotic count per high-power fields (HPFs) to a standardized area measured in mm^2^ addressing the well-known variability in field size across different microscopes from areas with highest mitotic density, even if these are found on different slides [2]. Lastly, the 5th edition of WHO classification emphasizes the role of diagnostic and predictive immunohistochemical biomarkers that will be discussed in detail in the present review. Additionally, it expands to transcriptome [24] and pangenomic analyses [25], as well as methylation profiling which may provide prognostic information [26], highlighting their emerging role in the molecular risk stratification of ACCs [27–29].

## Diagnostic approaches in ACC

**Fig. 1:**
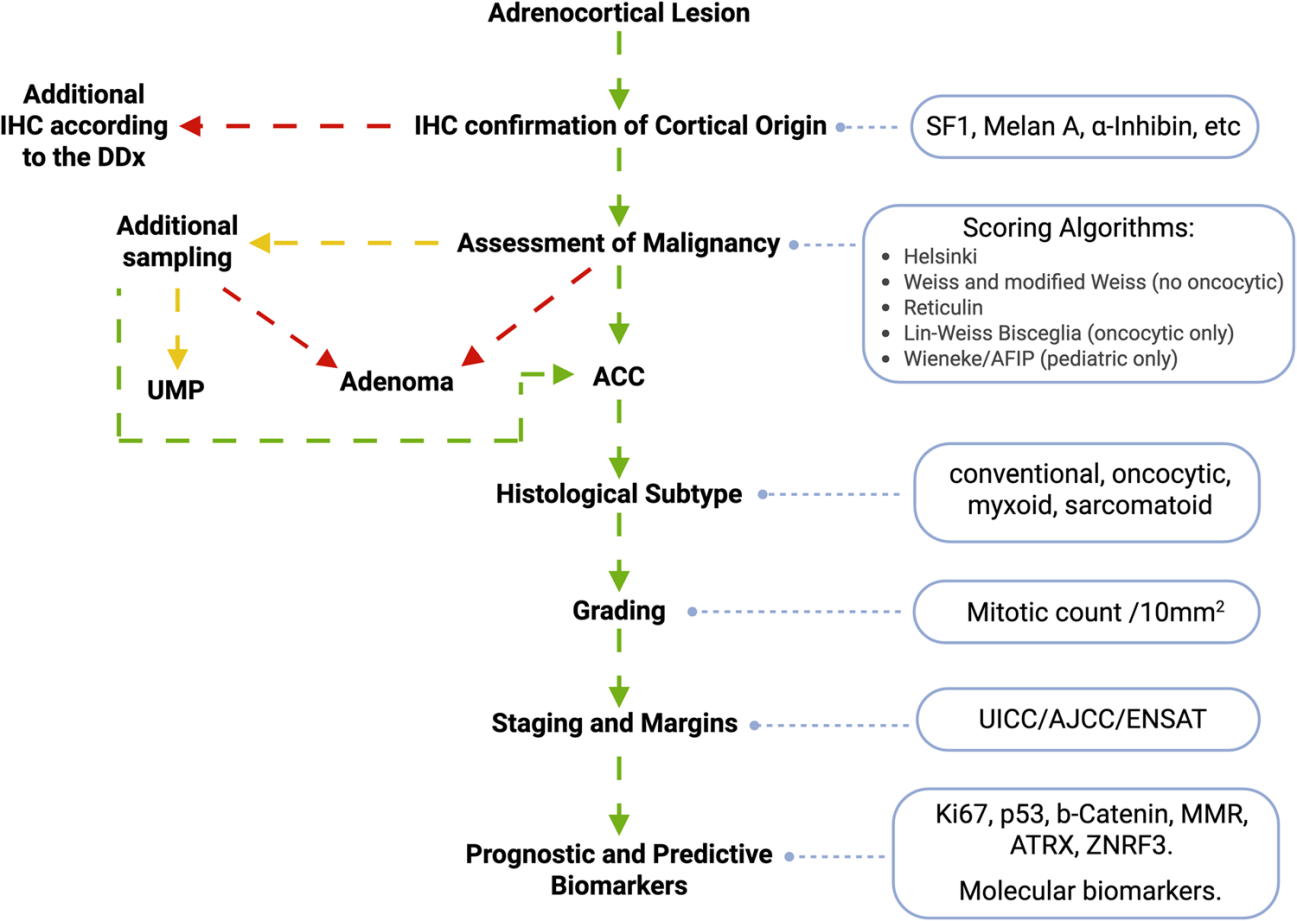
Diagnostic algorithm for the microscopical assessment of ACC. The arrows are color-coded: green refers to positive results, yellow to doubtful results, and red to negative results. IHC, immunohistochemistry; DDx, differential diagnosis; UMP, uncertain malignant potential; ACC, adrenal cortical carcinoma

A practical diagnostic algorithm is shown in Fig. 1.

### General macroscopy

ACC often presents as a large solitary adrenal mass, with a mean size of about 11 cm (range 1.6 to 30 cm) [5] and weighs around 350 g (range 4 to 3500 g) [30, 31].

Most ACCs are surrounded by a prominent fibrous capsule, and the macroscopic assessment of capsular integrity is important for diagnostic and prognostic purposes [32]. The demonstration of capsular invasion is also a key criterion in several multifactorial diagnostic scoring systems [33–35]. In addition to the identification of capsular invasion, generous sampling of the tumor capsule also allows for an adequate assessment of other types of invasion such as vascular and lymphatic “sinusoidal” invasion [32], which must be evaluated at or beyond the capsular edge [2, 32]. It is important to keep in mind that the probability of finding invasive foci is largely dependent on the extent of sampling; therefore, adequate tissue sampling should prioritize the tumor periphery and capsule over central tumor areas [32]. If evident, tumor extension beyond the tumor capsule into peri-adrenal soft tissues, large veins, or nearby organs should be promptly documented, as these aspects define stage III and stage IV disease [36]. Lastly, inking the surgical specimen before sectioning and careful assessment of tumor margins should always be performed.

On the cut surface, ACC usually presents as a vaguely nodular, yellowish-tan mass, eventually interspersed with areas of hemorrhage and necrosis. Nonetheless, a certain degree of heterogeneity is frequently observed, as a direct reflection of the diverse cellular composition and histological patterns intermingling within a single lesion. Therefore, accurate and exhaustive sampling of the tumor is recommended in order for it to be representative of its wide tumor heterogeneity.

**Fig. 2:**
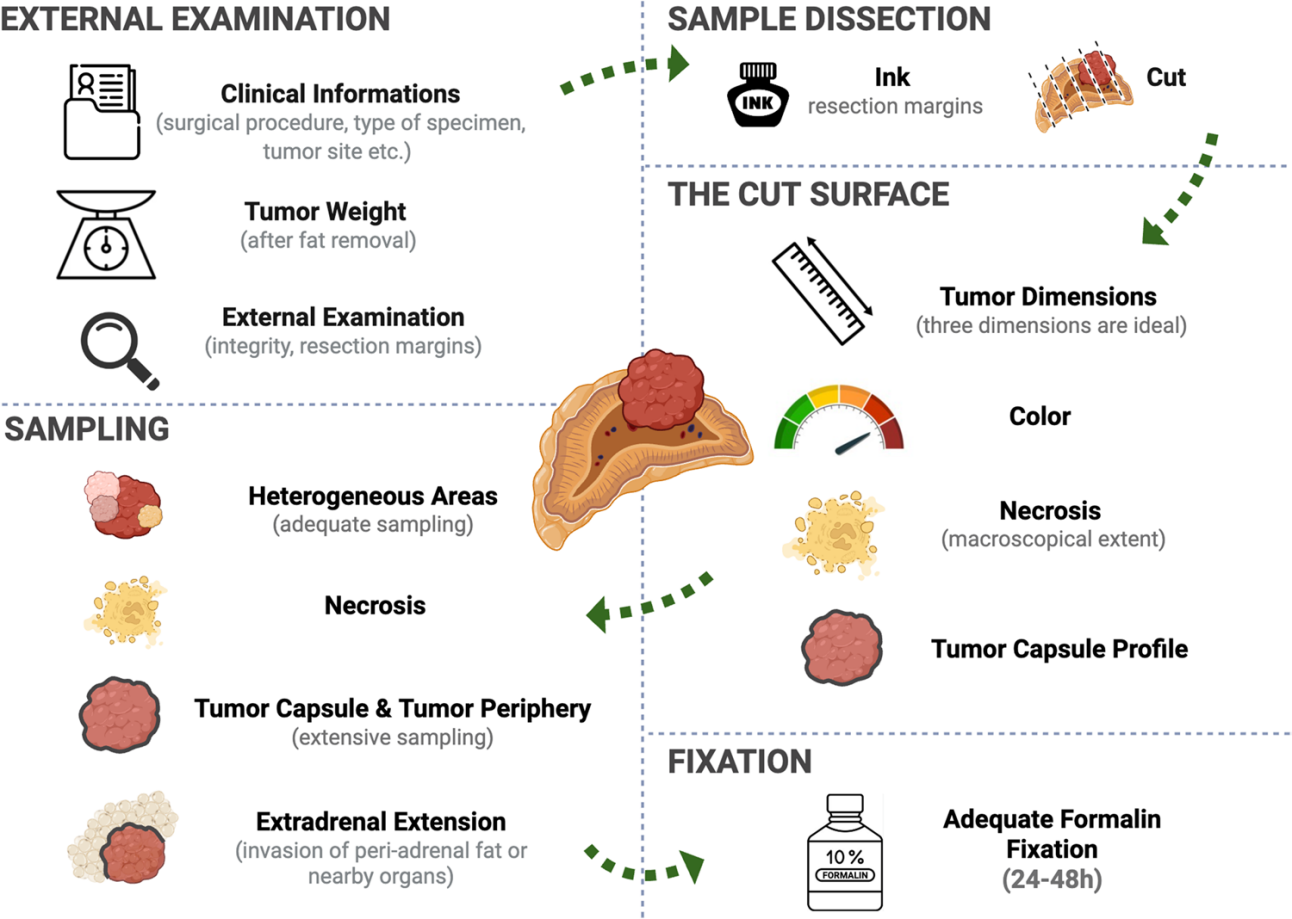
Overview of the macroscopic assessment process

An overview of the macroscopic assessment process is summarized in Fig. 2.

### General histopathology

**Fig. 3:**
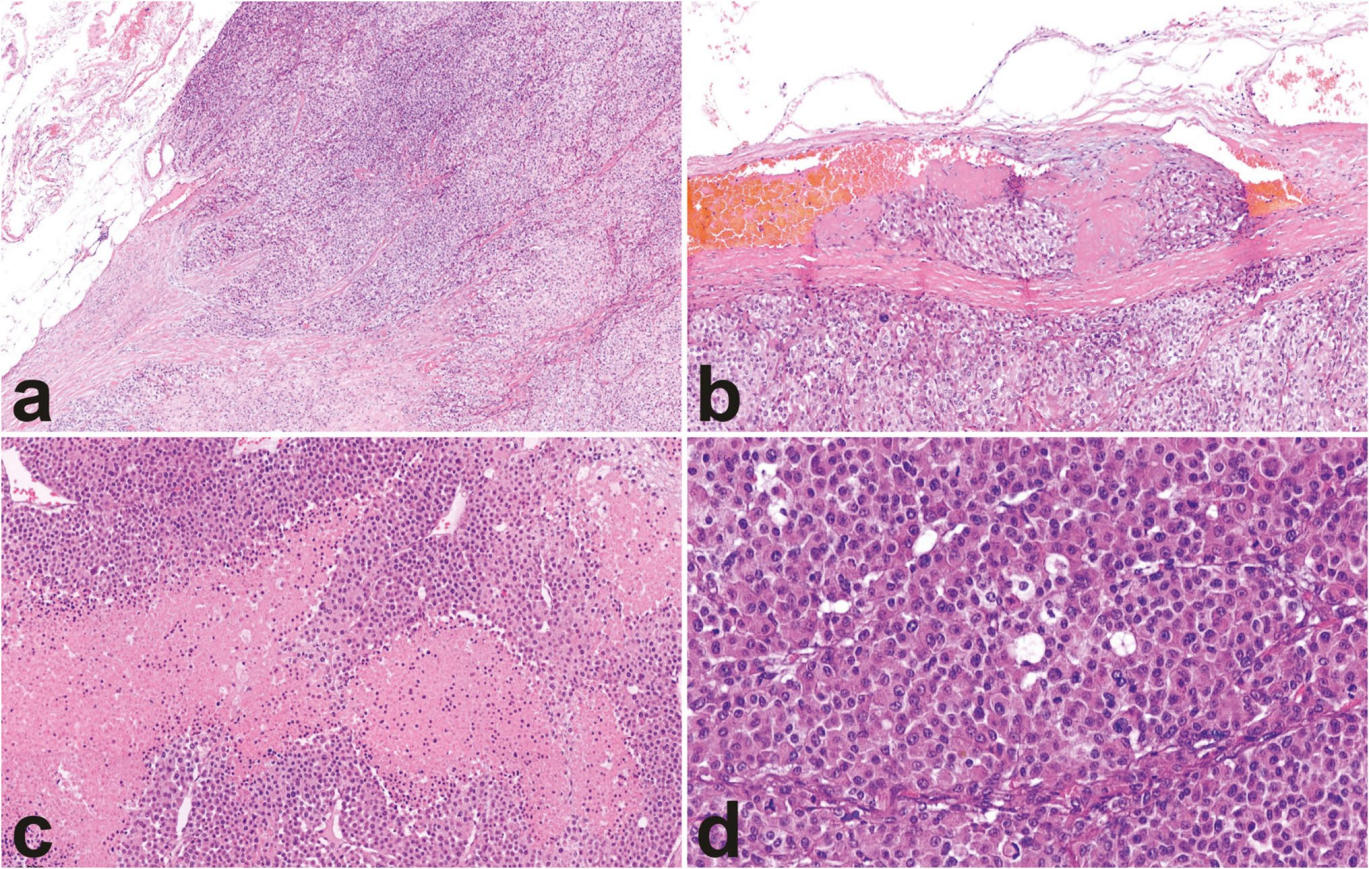
Microscopic features of ACC. Microscopic features suggestive of malignancy include capsular invasion and extracapsular extension (**a**, 10×), as well as angioinvasion, defined by tumor cells associated with fibrin thrombi in capsular (**b**, 20×) or extracapsular vessels. Other features associated with malignancy are coagulative necrosis (**c**, 25×), elevated mitotic count, and atypical mitoses (bottom left, a tripolar mitotic figure) (**d**, 40×)

Capsular invasion, despite its importance, has currently no universally accepted definition. Some authors regard any breach of the capsule as indicative of invasion, while others require full-thickness penetration to meet this criterion [32]. Moreover, its identification can be challenging due to irregularities in the capsule and the presence of fibrous interconnecting septa extending into the tumor. In contrast, direct invasion into peri-adrenal fat or adjacent organs constitutes definitive evidence of malignancy, as it reflects tumor extension beyond the adrenal capsule (Fig. 3a). In cases where the tumor completely breaches the capsule, it remains unclear whether a stromal reaction within the periadrenal fat is necessary to confirm periadrenal fat invasion, or if the mere presence of tumor cells within the periadrenal fat (with absent stromal response) should be considered sufficient evidence of such invasion.

Vascular invasion is assessed at the intersection of the tumor and adrenal capsule or beyond the adrenal capsule and should be distinguished in angioinvasion and sinusoidal invasion [2]. Even though gross or clinically apparent large vessel involvement has become an uncommon finding [32], data guiding the assessment of microscopic angioinvasion remain limited [2]. Recently, the most reliable histopathologic criterion for diagnosing microscopic angioinvasion has been tumor cell infiltration through a vessel wall accompanied by thrombus or a fibrin-tumor complex, or the presence of intravascular tumor cells intermixed with platelet thrombus or fibrin (Fig. 3b) [32, 37]. On the other hand, sinusoidal invasion is variably interpreted either as the presence of tumor cells within thin-walled vascular spaces inside the tumor or, more in line with current guidelines for pathology reporting [2, 32], as the invasion of lymphatic vessels at the periphery of the tumor. The lack of standardized definitional criteria contributes to the equivocal interpretation of sinusoidal invasion and makes it challenging to distinguish true invasion from artifacts caused by surgical specimen handling.

In line with the heterogeneity at macroscopy, ACC displays striking microscopic variability, which is often found in combination within the same tumor, both in terms of tumor architecture and cytological features. The most common pattern is broad trabecular growth, followed by alveolar or large nested, and solid/diffuse arrangements. The presence and extent of a solid or diffuse growth pattern should be noted, as it constitutes one of the criteria in the Weiss scoring system for ACC diagnosis [33]. Less frequently, pseudopapillary and storiform patterns may be observed. Despite this architectural heterogeneity, a unifying feature across all patterns is the loss of the well-organized alveolar architecture characteristic of non-neoplastic adrenal cortex. This architectural disarray serves as a valuable diagnostic clue as it can be demonstrated by the loss of the reticulin framework on silver stain-based histochemistry [5].

Most ACCs are composed of eosinophilic (lipid-poor) tumor cells, which may occasionally exhibit granular cytoplasm. Less frequently, ACC demonstrates clear (lipid-rich) cells, where the lipid content may be diffusely distributed within the cytoplasm or organized in a single vacuole displacing the nucleus, imparting a sort of “signet-ring” appearance. Nuclear atypia, pleomorphism, and hyperchromasia are almost invariably present. Notably, nuclear atypia may also occur in benign adrenal cortical lesions and is therefore a nonspecific feature. In contrast, the presence of one or more centrally located, prominent nucleoli is more characteristic of ACC and constitutes a key diagnostic criterion in the Weiss scoring system. The extent of nuclear pleomorphism can vary significantly within the same lesion and may include bizarre, multinucleated cells or, more rarely, rhabdoid features. In this context, the nuclear features of ACC are generally equivalent to grade 3 (prominent nucleoli visible at 100× magnification) or grade 4 (marked pleomorphism with anaplasia) according to Fuhrman grading criteria for renal cell carcinoma [38].

The presence of coagulative tumor necrosis is another important morphological feature to consider (Fig. 3c). When present, it is typically extensive, broad, and confluent, often exhibiting a comedo-like pattern. However, it may occasionally appear as punctate or focal, which increases the risk of it being overlooked, particularly in cases of suboptimal sampling. In terms of diagnostic and prognostic implications, necrosis is generally assessed as present or absent, whereas no studies have investigated, so far, the possible impact of the evaluation of the extent of necrosis, whenever present, in the characterization of ACC. A main limitation is related to the absence of clear and widely accepted definitions for focal vs. extensive necrosis, at variance with other tumor settings (i.e., sarcomas).

Other two parameters that are strongly associated with malignancy and are integrated in the diagnostic algorithms for ACC are the increased mitotic activity and the presence of atypical mitoses. The cutoff for mitotic index is defined as > 5 mitoses/10 mm^2^ for adults [2] and > 15 mitoses per 4 mm^2^ (20 HPF) for the pediatric patients [39, 40]. However, it is worth noting that in most studies of the available literature on the diagnostic and prognostic impact of mitotic index, mitotic count is expressed in 50 HPF rather than in 10 mm^2^, the latter being a strong recommendation of the last WHO classification scheme only. Therefore, future studies are needed to validate or refine clinically relevant cutoff values of mitotic index expressed in mm^2^. Atypical mitotic figures suggest underlying chromosomal abnormalities such as aneuploidy and are therefore regarded as a hallmark of malignancy, even when only a single, yet unequivocal, atypical mitotic figure is identified (Fig. 3d).

Lastly, tumor stroma may be characterized by intersecting fibrous bands and may display foci of dystrophic calcifications, which can be detected in up to 20% of cases. Lipomatous or myelolipomatous metaplasia can occur, while metaplastic bone formation is rarely seen. It is interesting to note that a lymphocytic inflammatory infiltrate can also be present at tumor periphery or intratumorally. Recently, it has been demonstrated that steroid production in ACC, in particular cortisol secretion as demonstrated by the expression of CYP17A and CYP11B1, significantly interferes with the tumor immune microenvironment, with special reference to the presence of inhibitory *T*_reg_ lymphocytes [41].

### Histological subtypes

**Fig. 4:**
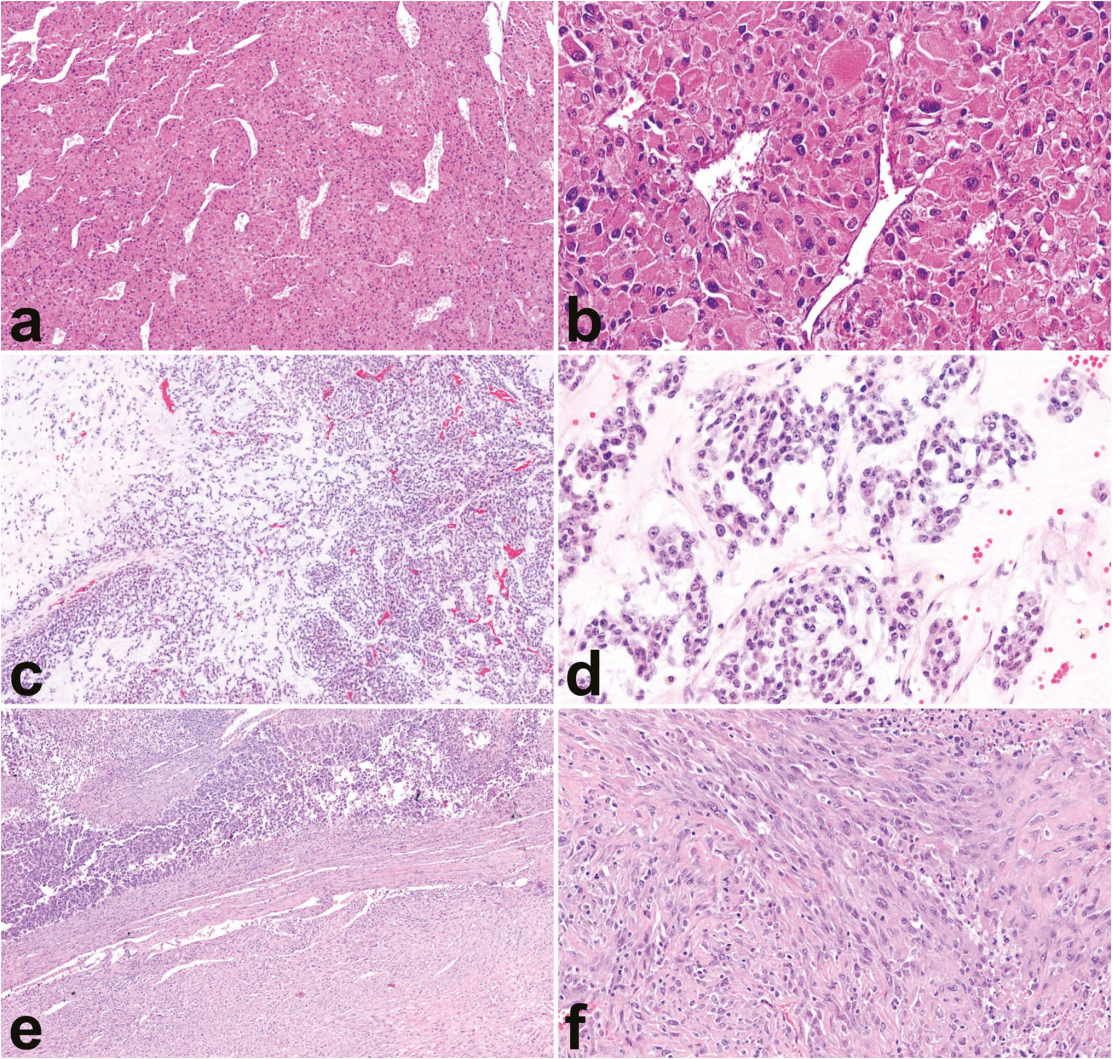
Histological subtypes. The oncocytic subtype is characterized by a predominant diffuse growth pattern (**a**, 10×) and large eosinophilic cells with abundant granular cytoplasm and nuclear atypia with prominent nucleoli (**b**, 40×). The myxoid subtype features neoplastic cells arranged in cords, thin trabeculae and microcysts admixed with variable amount of extracellular mucin (**c**, 10×) and less pronounced cytologic atypia (**d**, 40×). Lastly, the sarcomatoid subtype is characterized by a storiform architecture (**e**, 10×) composed by spindle cells featuring sarcomatoid appearance (**f**, 40×). Conventional ACC component may be present (**e**, 10×, upper half of the image). In the absence of the conventional component, differential diagnosis with pure sarcomas may be not always possible

In addition to the above-described conventional type of ACC, three histological subtypes are recognized, in the descending order of frequency: oncocytic, myxoid, and sarcomatoid [42] (Fig. 4). It is noteworthy that the WHO classification 5th edition adopts the term “sub-type” instead of the previously used “variant,” aiming to distinguish morphological categories (former) from genetic alterations (latter) [2].

These morphological patterns may blend with conventional features to a varying degree, ranging from complete absence to their prevalence, or even complete replacement of the conventional morphology.

The most common is the oncocytic subtype, defined by the presence of oncocytic cells comprising more than 90% of the tumor mass [34, 43]. These cells are large and characterized by their abundant, intensely eosinophilic, granular cytoplasm, which distinguishes them from the eosinophilic cells seen in conventional ACC. Another distinguishing feature of oncocytic ACCs lies in their consistent presentation of prominent nucleoli and a diffuse growth pattern, regardless of their underlying biological behavior.

The myxoid subtype is defined by the presence of a variable amount of extracellular myxoid-like material [44, 45], within which neoplastic cells are arranged in tiny trabeculae, cords, and/or microcysts. Importantly, myxoid changes alone are not diagnostic of malignancy.

Finally, the sarcomatoid subtype represents the least common form of ACC. It is characterized by mesenchymal differentiation in the context of a recognizable cortically derived carcinomatous component [46–48]. In the absence of the latter, these tumors can be indistinguishable from adrenal sarcomas [49, 50] if not for the presence of an even focal adrenal cortical marker expression. Importantly, this subtype has not been reported in the pediatric population.

### A critical reappraisal of scoring systems and diagnostic algorithms

The diagnosis of ACC relies on multiparametric scoring systems which variably integrate different histopathological features such as evidence of invasion, architectural and cytological features, mitotic activity, and the presence of necrosis [5, 31, 34, 35, 51, 52] (Table 1). Unfortunately, nearly all of these histopathological parameters are laden with interpretive complexity, posing significant challenges in routine diagnostic practice. Therefore, the integration of multiple algorithms is particularly advisable in cases of adrenocortical lesions where overt clinical or morphological indicators of malignancy are lacking.

**Table 1:** Main scoring/diagnostic algorithms in ACC

Parameter	Weiss score	Modified Weiss (Aubert)	Helsinki score	Reticulin algorithm	Lin-Weiss Bisceglia	Wieneke/AFIP criteria
Applicability	Adult ACC (except oncocytic subtype)		All adult + pediatric ACC		Only adult oncocytic subtype ACC	Only pediatric ACC
Capsular invasion	1	1	**-**	**-**	Minor criterion	1 (+ 1 extra if extra capsular extension)
Angioinvasion	1	**-**	**-**	Additional parameter	Major criterion	1 (+ 1 extra if invasion of vena cava)
Sinusoidal (lymphatic invasion)	1	**-**	**-**	**-**	Minor criterion	**-**
Nuclear atypia (grade 3–4^#^)	1	**-**	**-**	**-**	**-**	**-**
Clear cells < 25%	1	2	**-**	**-**	**-**	**-**
Diffuse architecture > 30%	1	**-**	**-**	**-**	**-**	**-**
Coagulative necrosis	1	1	5	Additional parameter	Minor criterion	1
Mitotic count > 5/10 mm^2^	1	2	3	Additional parameter	Major criterion	1 (if > 15/20 HPF)
Atypical mitotic figures	1	1	**-**	**-**	Major criterion	1
Ki67 index (as %)	**-**	**-**	Numeric value of Ki67%	**-**	**-**	**-**
Disruption of reticulin framework	**-**	**-**	**-**	Main parameter	**-**	**-**
Size > 10 cm	**-**	**-**	**-**	**-**	Minor criterion	1 (if > 10.5 cm)
Weight > 200 g	**-**	**-**	**-**	**-**	Minor criterion	1 (if > 400 g)
Cutoff score for Malignancy	≥ 3	≥ 3	> 8.5	Main + 1 of the additional parameters	1 major criterion *UMP: if 1 or more minor criteria only is present*	> 3 *UMP: 3* *Benign: 0–2*
Main advantages	Mostly validated; no special stains required	No special stains required	Easy to assess, good reproducibility		Most validated in oncocytic sub-types; no special stains required	No special stains required
Main disadvantages	Poor reproducibility of some parameters; risk of overestimation for the oncocytic subtype; risk of underestimation for the myxoid subtype	Risk of overestimation for the oncocytic subtype; risk of underestimation for the myxoid subtype	Needs Ki67 stain	Requires reticulin stain; need of larger validation; sites of degeneration may be a pitfall	Needs tumor weight; only applicable to oncocytic adult subtype	Not 100% sensitive nor specific; Needs tumor weight

Legend. Numbers refer to points; *UMP*, uncertain malignant potential; #according to Fuhrman’s grading of renal cell carcinoma

The Weiss system, proposed in 1989, is the most widely adopted and validated algorithm, made up of nine histopathological, purely morphological parameters.

Each parameter accounts for 1 point, for a maximum of 9 points, and malignancy was defined by a score of ≥ 3 points [33]. Given that some of these parameters are highly subjective and poorly reproducible [53] and aiming to increase reliability, a modified Weiss score was proposed by Aubert in 2002. This simplified system eliminated the four least reproducible parameters (angioinvasion, sinusoidal invasion, nuclear atypia, and diffuse architecture) and doubled the weight of other parameters (extent of clear cell and mitotic count), for a maximum of 7 points, with malignancy defined by a score of ≥ 3 points [35]. Both scores are inapplicable in pediatric ACCs, as the specificity of the Weiss score has demonstrated to be low, generally overestimating the malignant potential [40]. Similarly, both scores overestimate malignancy in the adult oncocytic subtype but underestimate malignancy in the myxoid subtype. In fact, in oncocytic neoplasms, 3 Weiss parameters (diffuse growth, eosinophilic cytoplasm, and nucleoli) are invariably present also in oncocytic adenomas, whereas in myxoid cases, the diffuse growth pattern, lympho-vascular invasion, and nuclear atypia may be absent or challenging to evaluate, thereby increasing the risk of underdiagnosing a malignant lesion [45].

To address the diagnostic challenges posed by the oncocytic subtype, a dedicated algorithm, the Lin-Weiss-Bisceglia (LWB) system, was introduced in 2004. This model was specifically designed to overcome the limitations of traditional scoring systems when applied to oncocytic adrenocortical tumors. The LWB algorithm is based on three major and four minor criteria for malignancy, and a diagnosis of malignancy is established when at least one major criterion is present. In contrast, tumors exhibiting at least one minor criterion in the absence of major ones are classified as having uncertain malignant potential (UMP). Key limitations of this system include its applicability exclusively to the oncocytic subtype and its reliance on tumor weight, a parameter not consistently available [34].

More recently, the Helsinki score has emerged as a streamlined and effective diagnostic tool. This algorithm relies on just three parameters: mitotic count, the presence of tumor necrosis, and the Ki-67 proliferative index, specifically measured in the most proliferative area of the tumor, with a final score > 8.5 supporting a diagnosis of ACC [52]. In comparative analyses, the Helsinki score has demonstrated superior predictive accuracy for malignancy over the Weiss system [49], with 100% sensitivity and 99.4% specificity for identifying a metastatic potential. Furthermore, a threshold of 28.5 has been validated as a prognostic indicator of overall survival in a large cohort study [54]. Additionally, the system has been extensively validated across independent cohorts, including both conventional ACC and its histological subtypes [55], as well as in the pediatric population (at the cutoff score of 24) [56].

**Fig. 5:**
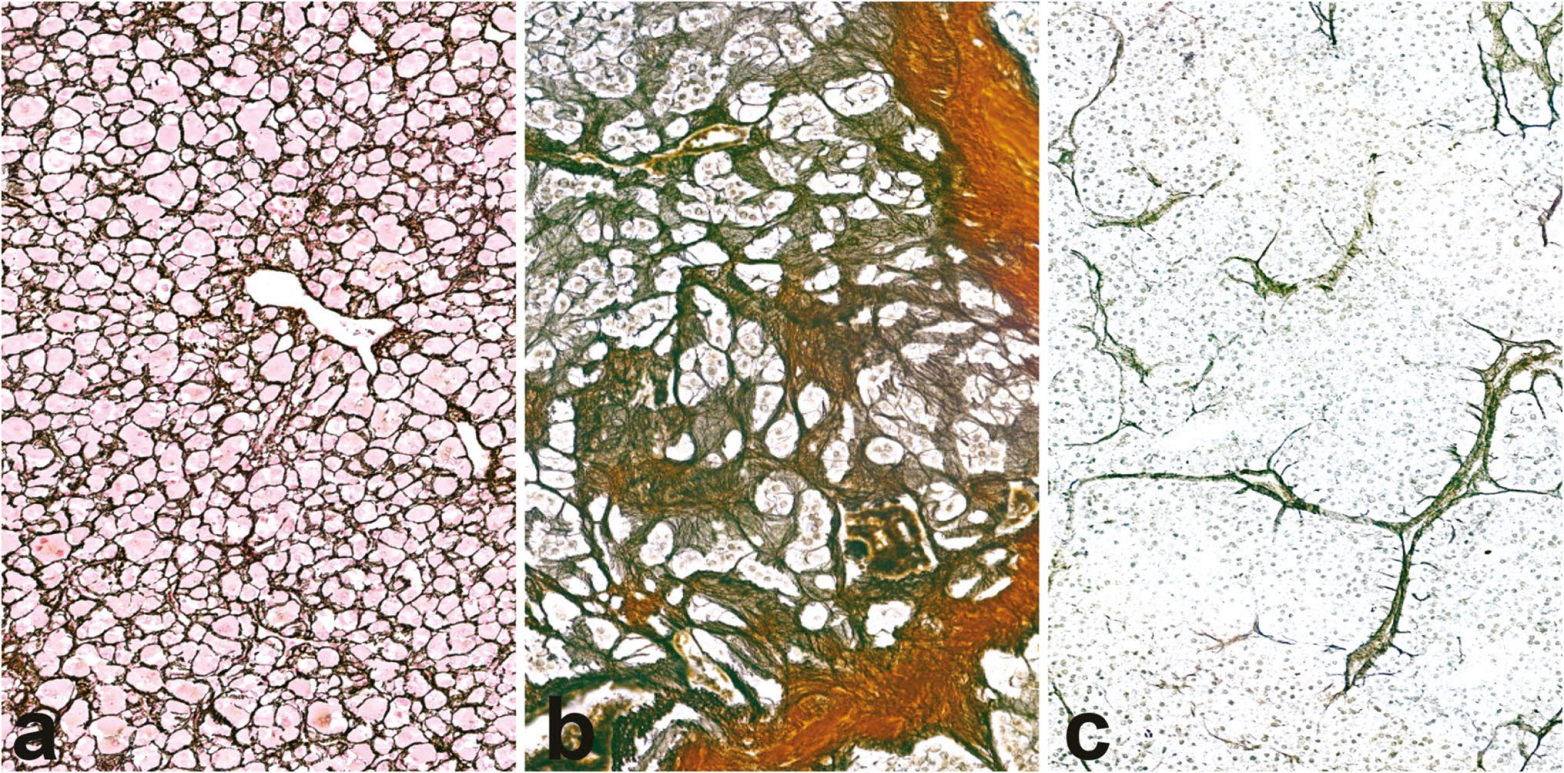
Patterns of reticulin stain in ACC. Normal reticulin pattern in normal adrenal cortex features a regular alveolar architecture (**a**, 20×). In contrast, ACC displays an altered reticulin pattern, manifesting either in form of “qualitative changes” or “quantitative changes”. Qualitative changes (**b**, 20×) retain the overall reticulin network but show irregular fiber thickness encasing small groups or individual tumor cells. Quantitative changes (**c**, 20×) are defined by fiber disruption determining a loss of continuity within the reticulin framework

The reticulin algorithm [31] is predicated on the observation that ACC displays a significant degree of architectural disarray, reflected by qualitative and/or quantitative alterations in the reticulin network (Fig. 5) [37], as demonstrated by silver-based staining methods [31]. For the diagnosis of ACC, the algorithm requires the demonstration of an altered reticulin framework combined with the presence of at least one of three additional parameters, namely necrosis, increased mitotic index, and vascular invasion. The reticulin algorithm has 100% sensitivity and specificity in distinguishing cases coded as benign or malignant by the Weiss system, but it is easier and more reproducible, and its accuracy has been validated in multiple independent cohorts [55, 57], including the pediatric population [56]. Additionally, its potential for objective quantification through computerized morphometric analysis [58] suggests possible future integration into computational pathology–supported diagnostic tools.

The Wieneke/AFIP Scoring System, introduced in 2003, was specifically designed for the pediatric population [39]. It encompasses nine histological criteria, with a cumulative score exceeding 3 considered indicative of malignancy. Increasing scores correlate with worse overall and disease-free survival outcomes [59]. Despite its utility, this system is limited by its exclusive applicability to pediatric cases and by the incomplete sensitivity and specificity of its criteria.

## ACC histopathological tumor grading

According to the WHO classification 5th edition, ACCs should be graded using a two-tiered system based on the mitotic count. ACC is therefore classified as low-grade ACC (mitotic count is ≤ 20 mitoses per 10 mm^2^) or high-grade (mitotic count is > 20 mitoses per 10 mm^2^) [2, 37]. This grading system has originally been proposed by Weiss in 1989 [33] and has since been validated in several adult patients’ cohorts [32, 37, 60] proving to carry a significant prognostic value [33, 37]. Interestingly, recent studies in two independent cohorts have suggested that a cutoff of 10 mitoses per 10 mm^2^ may offer improved prognostic performance in ACC [37, 61], but broader validation is needed.

Importantly, no formal grading system has yet been established for the pediatric population, as an optimal mitotic count threshold for stratification remains to be determined through large-scale clinical studies.

Lastly, although the Ki-67 proliferation index has been shown to have prognostic significance [59], it has not been adopted as a grading tool in the current WHO classification, as such proliferation indices represent continuous variables in tumor biology, rather than fixed/static cutoff points [2].

## A practical approach to immunohistochemical markers

Immunohistochemical markers in ACC serve three main purposes: confirming the adrenocortical origin (Table 2), assisting in the distinction between benign and malignant adrenal cortical lesions, and providing prognostic information.

**Table 2:**
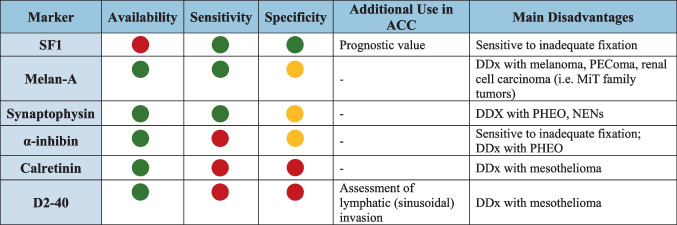
Immunohistochemical markers of primary adrenocortical origin

Legend. Markers are color coded: green refers to favorable characteristics, yellow indicates potential issues while red indicated major issues. *DDx*, Differential diagnosis; *PHEO*, phaeochromocytoma; *NEN*, neuroendocrine neoplasms

The former two are discussed below, while the latter is addressed in the dedicated section below.

**Fig. 6:**
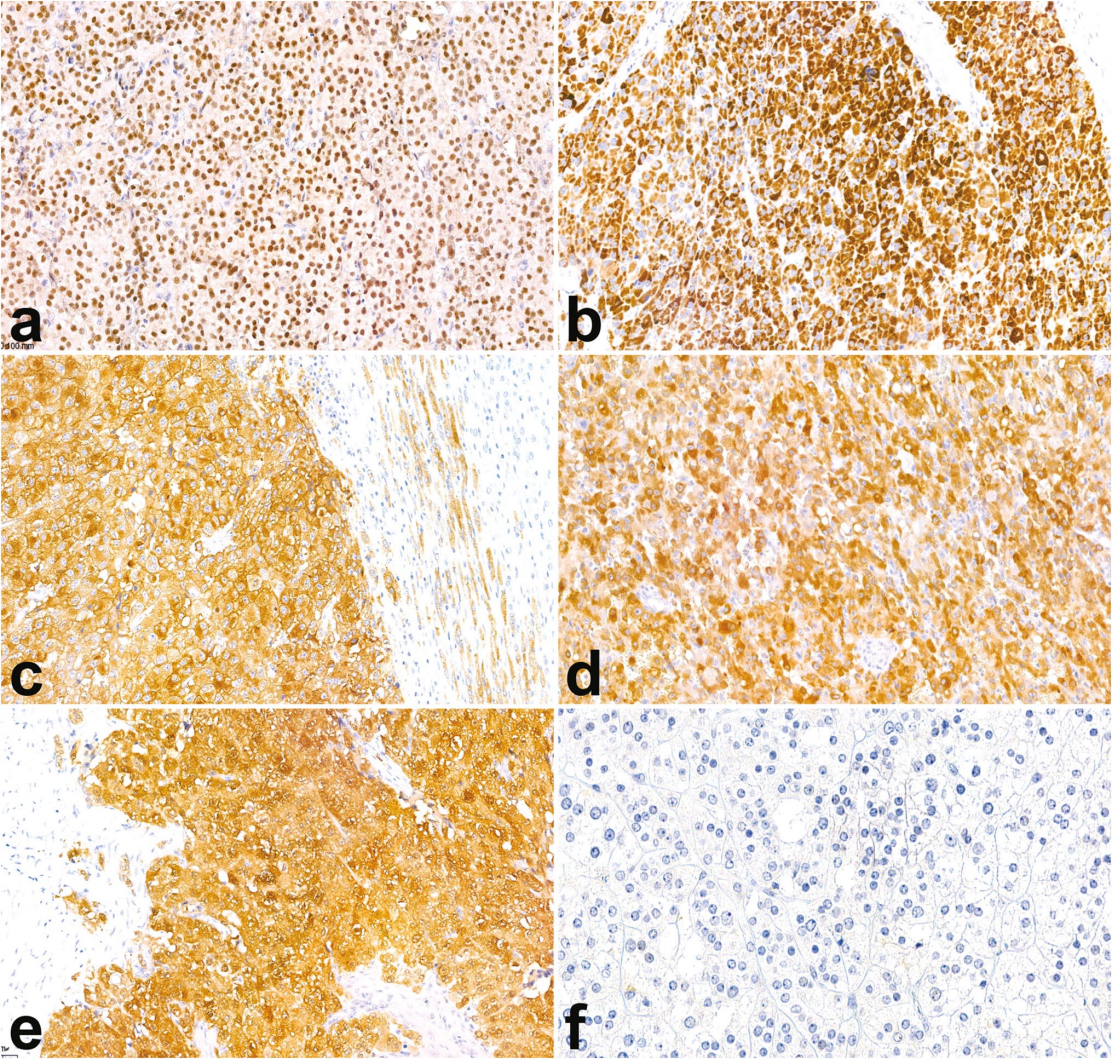
Immunohistochemical markers of adrenocortical origin. SF1 nuclear positivity is the most reliable marker confirming the cortical origin (**a**, 20×). Other markers of cortical origin, although less specific, include the cytoplasmic expression of Melan A (**b**, 20×), synaptophysin (**c**, 20×), α-inhibin (**d**, 20×), and calretinin (**e**, 40×). Conversely, cytokeratin (here the AE1/AE3 clone) is usually negative in ACC (**f**, 40×)

The most reliable biomarker to confirm the adrenocortical origin is SF1 [62], a nuclear receptor involved in the regulation of steroidogenesis [63]. SF1 exhibits nuclear staining (Fig. 6) and demonstrates excellent diagnostic performance, with up to 100% specificity and 95% sensitivity [64, 65]. However, despite its value, antigenicity may be lost in sub-optimally fixed specimens, and more broadly, the antibodies are not readily available in all centers.

**Fig. 7:**
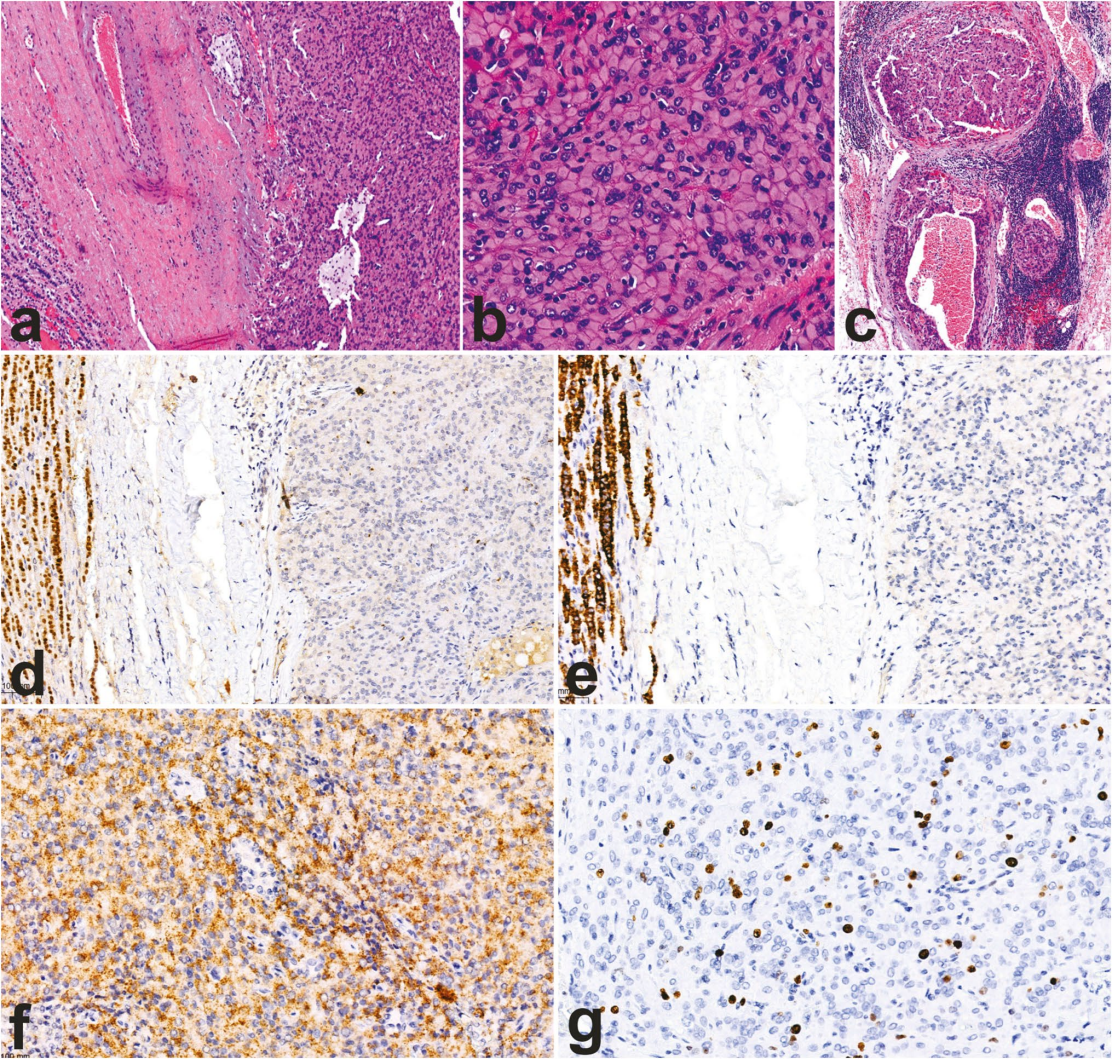
Intra-adrenal PEComa mimicking ACC. The intra-adrenal lesion (**a**, 5×; bottom left, peritumoral adrenal cortex) is composed of epithelioid eosinophilic cells arranged in a vaguely trabecular architecture (**b**, 40×). Metastasis to a periadrenal lymph node is shown in (**c**, 10×). In contrast to the adjacent normal adrenal cortex, the tumor shows the loss of SF1 expression (**d**, 25×). The present case was also negative for Melan A (**e**, 25×). Further immunohistochemical analyses revealed diffuse cathepsin K positivity (**f**, 25×) and a moderately low proliferative index, with Ki-67 staining around 10% (**g**, 25×)

Cytoplasmic markers with lower specificity for adrenocortical origin such as Melan-A, synaptophysin, α-inhibin, calretinin, and D2-40 are more broadly available [62], but their diagnostic performance varies: Melan-A and synaptophysin offer high sensitivity but moderate specificity, α-inhibin shows low sensitivity with moderate specificity, and calretinin and D2-40 are both the least sensitive and specific among them. It should be emphasized that some of the abovementioned markers may also be expressed by neoplasms that closely mimic ACC: Melan-A can be expressed by melanoma [66], PEComa [67], and renal cell carcinoma (RCC) [68]; synaptophysin is positive in paragangliomas [62] and neuroendocrine neoplasms; α-inhibin is also positive in a subset of paragangliomas and various non-adrenal carcinomas [69]. In such contexts, employing a panel of adrenocortical markers alongside lineage-specific immunohistochemical stains tailored to the differential diagnosis can enhance diagnostic accuracy and reduce the risk of misdiagnosis. A case of primary adrenal malignant PEComa is illustrated in Fig. 7, as an example of an ACC mimicker.

If cortical origin is immunohistochemically confirmed, the presence of invasive growth or high-grade features warrants the diagnosis of ACC. Conversely, in front of low-grade features, the diagnosis of ACC should be challenged with that of adrenocortical adenoma and may require ancillary biomarkers [37, 62]. To this end, in addition to the histopathological features composing multiparametric scoring systems and the previously discussed reticulin silver stain, some immunohistochemical markers of malignancy have been proposed in adrenal cortical tumors. Among them, insulin-like growth factor 2 (IGF2) immunostaining can be utilized. With a juxtanuclear granular staining pattern, IGF2 has proven to be a specific marker for ACC ranking as the most reliable ancillary tool for distinguishing ACC from adenoma [2]. Additionally, p53 could also be employed, as the altered expression of this marker supports the diagnosis of ACC, as also discussed below. However, as this alteration is more typical of high-grade ACC, its diagnostic accuracy in the context of low-grade features is notably low [62].

## Pathological, immunohistochemical, and molecular prognostic markers

From a pathological standpoint, numerous histopathological features have been recognized as prognostically relevant. Capsular invasion has been recognized as an independent risk factor for mortality, and angioinvasion is emerging as one of the most powerful prognostic indicators in ACC [37], highlighting the critical importance of accurate identification of these features. More broadly, positive surgical margins were found to be an independent predictor of both shorter overall survival (*p* = 0.04) and recurrence-free survival (*p* = 0.03)[70], reinforcing earlier evidence demonstrating a significantly increased risk of mortality associated with margin involvement in multivariable analysis (*p* < 0.0001) [71]. Lastly, necrosis has generated particular interest as its presence has been shown to adversely affect both overall survival (*p* = 0.05) and disease-free survival (*p* < 0.001), emerging as the strongest adverse prognostic factor within the Weiss scoring system [72].

**Fig. 8:**
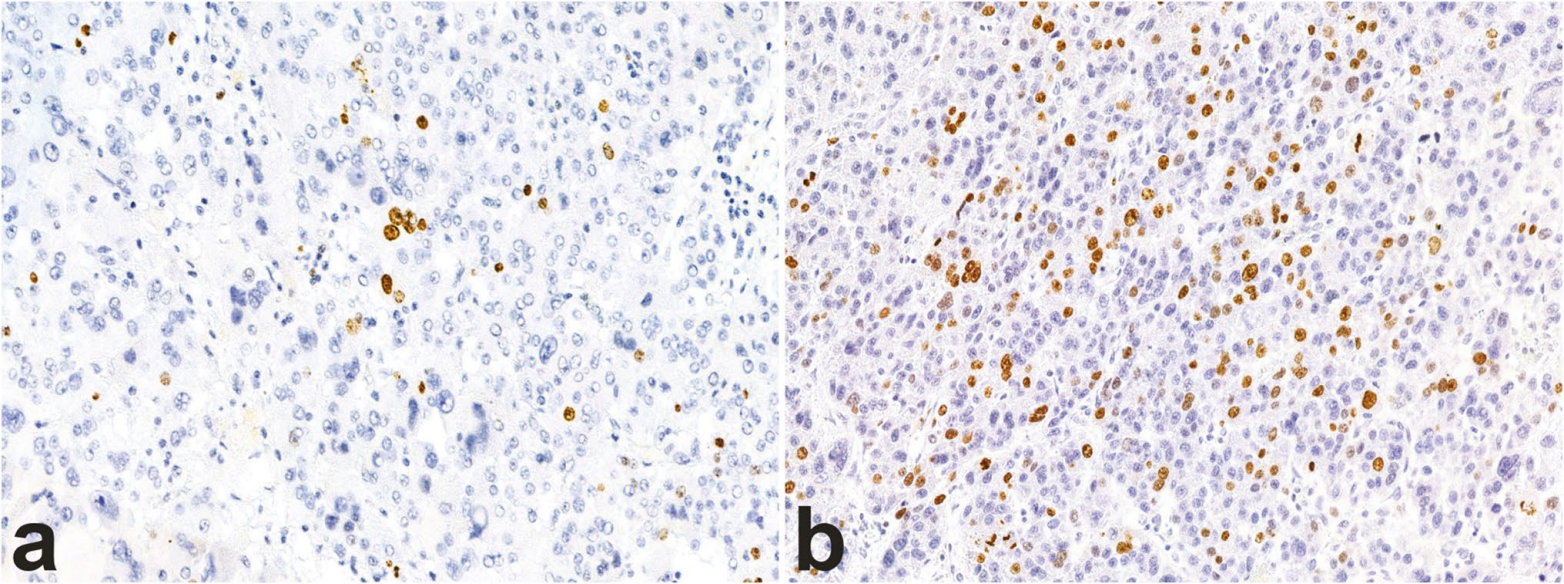
Heterogeneity of Ki-67 index in ACC. Ki-67 proliferation index may be lower than 10% in some cases (**a**, 10×) but in most cases is elevated (**b**, 10×)

Concerning immunohistochemical biomarkers, the most relevant is the Ki-67 proliferation index [59, 73] (Fig. 8), although a consensus on prognostic cutoff values has yet to be established. In this context, the development of diagnostic algorithms, whether based on manual counting or automated image analysis, may improve the standardization of its assessment [74], potentially helping to bridge this gap in the future. This would be particularly valuable given the index’s utility in guiding decisions regarding adjuvant mitotane therapy [22]. However, a main limitation in the application of Ki-67 in clinical practice is represented by low reproducibility, with special reference to the interpretation of the results that leads to poor inter-observer agreement [75]. The use of digital image tools has been claimed to represent a possible solution to implement reproducibility, but it needs appropriate settings, in particular to reduce the risk of overestimating the proliferation index [74].

Another proliferative marker, phosphohistone H3 (PHH3), has been implied to improve the accuracy of mitosis identification [61], whose important prognostic role has been discussed previously. However, its utility has not yet been fully validated.

**Fig. 9:**
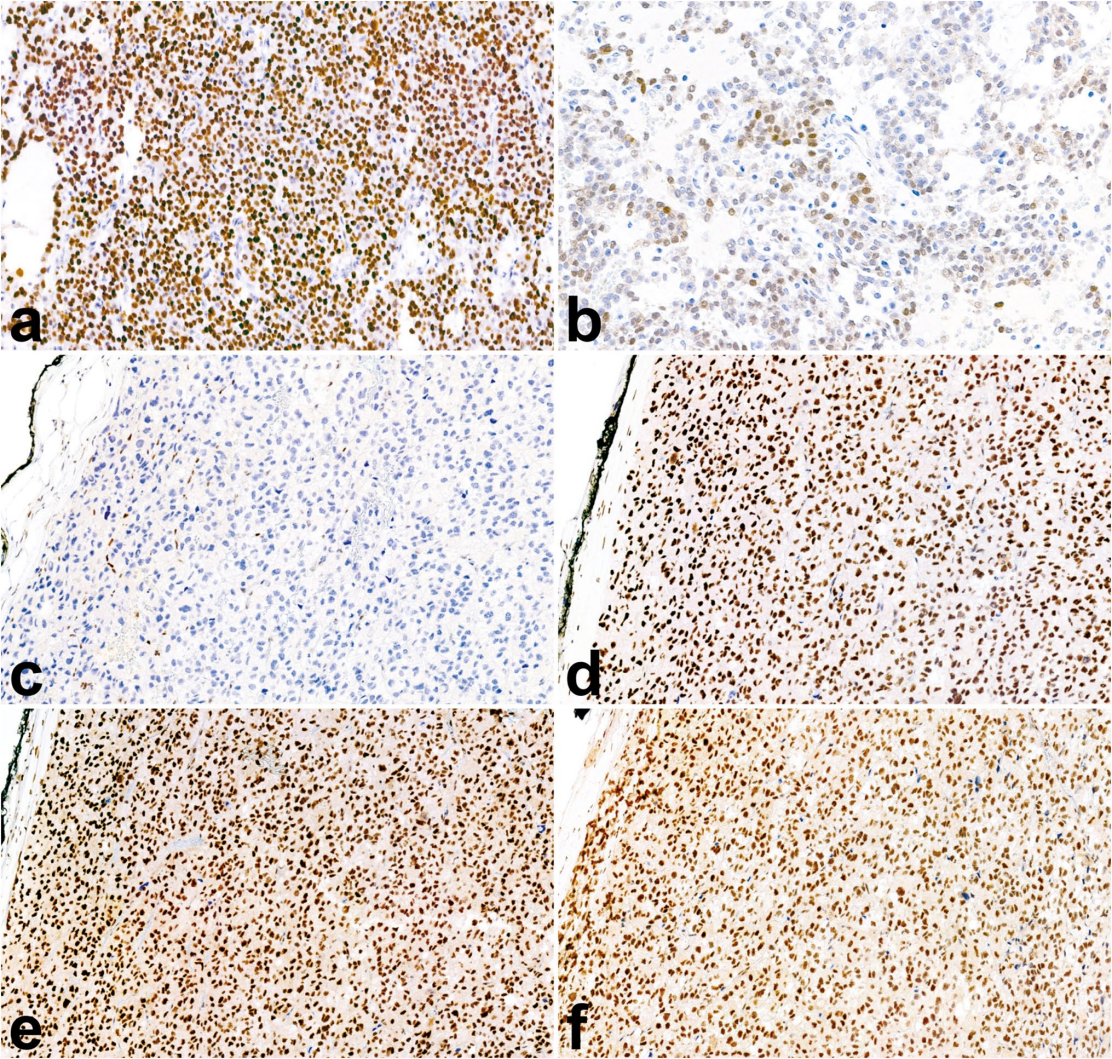
Immunohistochemical stains as surrogate molecular markers in ACC. Altered p53 expression presented as overexpression (**a**, 20×); altered β-catenin expression, as evidenced by aberrant nuclear positivity (**b**, 20×); mismatch repair deficiency (MMRd) evidenced by the loss of MSH6 protein expression, with intact internal control (**c**, 20×) and preserved MSH2 (**d**), MLH1 (**e**, 20×), and PMS2 (**f**, 20×) expression

P53 and β-catenin are recognized as key translational prognostic biomarkers, as altered expression of these proteins is frequently observed in carcinomas associated with high-risk molecular profiles. Aberrant p53 staining presents as either diffuse nuclear expression or complete absence, while altered β-catenin expression is marked by diffuse nuclear expression [62] (Fig. 9). Additional markers linked to poorer clinical outcomes include high expression of SF1 [62, 64] and loss of ATRX and ZNRF3 expression [76].

Moreover, immunohistochemical evaluation of mismatch repair (MMR) proteins (Fig. 9) and SDHB can aid in identifying underlying germline alterations associated with Lynch syndrome and SDH-related familial paraganglioma syndrome, respectively. Consequently, the application of these markers is recommended in all apparently sporadic cases of adrenocortical carcinoma [62]. Additionally, MMR proteins, together with PD-L1 immunohistochemistry, could help identify patients with susceptibility to immune-enhancing therapies [77].

Finally, the prognostic role of CYP11B1 and CYP11B2, known as immunohistochemical markers of steroid-secreting adrenocortical neoplasms [78] remains controversial in ACC [79].

Recent transcriptomic [80] and pan-genomic [27] studies have increasingly underscored the prognostic potential of molecular characterization in adrenocortical carcinoma (ACC). For instance, alterations in the *TERT* gene have been linked to unfavorable clinical outcomes, including metastatic progression and disease-specific mortality [81]. Similarly, methylation profiling has emerged as a valuable tool for predicting prognosis [26]. In this context, dysregulation of microRNAs, such as downregulation of miR-195 and overexpression of miR-483-5p, as well as hypermethylation of the GO/G1 Switch 2 (*G0S2*) gene, has all been associated with poorer outcomes and increased mortality risk [82, 83]. Furthermore, *RRM1* gene expression has gained attention as a predictive biomarker for response to adjuvant mitotane therapy in ACC while elevated CYP2W1 mRNA levels have been correlated with improved survival in patients receiving mitotane treatment [84].

A schematic overview of the key characteristics of the principal immunohistochemical and molecular biomarkers is shown in Table 3.

**Table 3:**
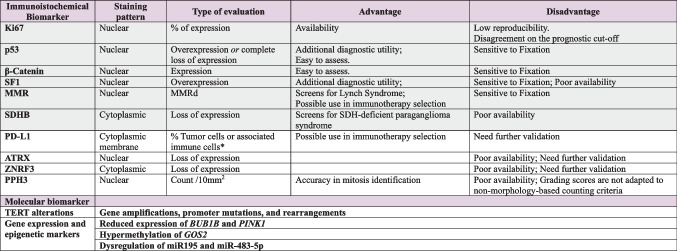
Prognostic immunohistochemical and molecular biomarkers in ACC

Legend. Light gray boxes indicate immunohistochemical markers with stronger validation and are thus the most recommended; *MMRd*, deficient mismatch repair protein function; *, no specific PD-L1 scoring criteria or cut-offs have been officially established to date

## Synoptic standardized pathology reporting

The pathological evaluation of ACC remains challenging, complex, and potentially ambiguous, and a standardized approach to the pathological evaluation of ACC would significantly enhance risk stratification for individual patients and would enable robust multinational translational research [32]. To this end, in 2021, the International Collaboration on Cancer Reporting (ICCR) convened an expert panel to review the pathological reporting of ACC and subsequently established a standardized dataset for ACC reporting, now available on the ICCR website (https://www.iccr-cancer.org/datasets/published-datasets/endocrine/adrenal-cortex/).

The dataset subdivides elements into core and non-core. Briefly, the core elements refer to data points deemed essential for clinical management, staging, or prognostication, and for which there is unanimous consensus among the expert committee. In contrast, non-core elements may hold clinical relevance but lack consistent validation or widespread implementation in routine patient management. The application of this scheme is strongly encouraged to implement standardization of pathological reporting and increased diagnostic reproducibility.

All these elements are summarized in Table 4.

**Table 4:** International Collaboration on Cancer Reporting (ICCR) dataset for pathology reporting of ACC. Data adapted from (https://www.iccr-cancer.org/datasets/published-datasets/endocrine/adrenal-cortex/)

	Core elements	Non-core elements
Clinical	Clinical information (e.g., symptoms, functionality, syndromes, prior therapy)	
	Operative procedure (open or laparoscopy)	
	Type of specimen submitted (also specimen other than adrenal gland should be identified)	
	Tumor site	
Macroscopical	Specimen integrity (intact or fragmented)	
	Tumor dimensions (largest single dimension)	Additional two dimensions
	Tumor weight (after adipose tissue and other organs are removed)	
Microscopical	Histological tumor type (according to the WHO)	
	Extent of invasion (invasion of extra-adrenal adipose tissue or nearby organs)	
	Tumor architecture (trabecular, alveolar, nested or diffuse)	
	Clear (lipid-rich) cells	
	Capsular invasion	
	Lymphatic (sinusoidal) invasion	
	Vascular invasion	
	Atypical Mitotic Figures	
	Coagulative tumor necrosis	Extent of necrosis
	Nuclear grade	
	Mitotic count and histological tumor grade	
	Ki67 proliferation index (measured on the area with the highest mitotic count)	
	Margin status	Distance of the tumor to the closest margin
	Lymph node status	Extra-nodal extension
	Histologically confirmed distant metastasis	
	Pathologic staging (UICC/AJCC)	
		Multifactorial scoring systems
		Ancillary studies (reticulin, SF1, NGS)
		Coexisting adrenal pathology (e.g., adenoma)

Legend. *UICC*, Union for International Cancer Control; *AJCC*, American Joint Committee on Cancer; *NGS*, next-generation sequencing

## Potential utility and limitations of artificial intelligence application in the field of ACC

In the field of ACC, as in many other medical fields, the role of artificial intelligence (AI) is rapidly expanding.

Considering that the CT scan represents a mandatory diagnostic tool used in patients with a clinical suspicion of adrenal mass, it is unsurprising that it has received considerable interest. In 2022, a Japanese retrospective single-center study used two methods (U-Net architecture and region-based convolutional neural network) to develop AI models to detect and classify adrenal masses. Although AI assistance was associated with improved sensitivity for less experienced radiologists, for an experienced physician, AI suggestion seemed to hamper performance [85]. Two preliminary works, both of which incorporated radiomics features, were presented. A retrospective Chinese multi-institutional study extracted radiomics features from different phases of contrast-enhanced CT images from 158 patients and developed an interpretable radiomics model which had superior diagnostic performance compared to two experienced radiologists (AUC model 0.92 vs. AUC radiologist 1 0.79, AUC radiologist 2 0.63) [86]. A larger retrospective European study analyzed 794 adrenal masses using texture analysis on unenhanced CT scans and showed that a radiomic-based DL algorithm was highly accurate in predicting the presence of malignant adrenal masses and specifically performed well in predicting ACC (AUC = 0.933, F1-score = 0.318, sensitivity = 96.4%, specificity = 83.9%) [87].

To the best of our knowledge, very few AI tools specifically developed for the diagnosis of ACC have been published. We found a single very recent study in the literature that applied pathomics analysis to ACC. In this study, a specific signature based on 5 features (related to cell density, chromatin characteristics, and staining intensity) was developed and integrated with clinical characteristics into a nomogram that proved to have prognostic impact in ACC [88].

The application of deep learning techniques to whole slide images in the context of ACC has likely been hindered by the rarity of the disease and consequent limited availability of digitized ACC histology slides, significant histological heterogeneity, which complicates model development, and intrinsic diagnostic difficulties with varying diagnostic interpretation even among experts which translate into an inability to obtain enough reliable ground truth annotations.

More broadly, ethical challenges persist in deploying these systems, as they have been developed in specific populations and this potentially may compromise model accuracy in other populations. Lastly, the limited explainability of current AI systems remains a key barrier to their clinical adoption. Addressing the “black box” nature of deep learning algorithms will require not only technical advances in interpretable modeling, but also sustained interdisciplinary collaboration between pathologists, computer scientists, and regulators, to ensure that future AI tools will be scientifically robust and clinically trustworthy.

## References

[1] Sharma E, Dahal S, Sharma P et al (2018) The characteristics and trends in adrenocortical carcinoma: a United States population based study. J Clin Med Res 10:636–640. 10.14740/jocmr3503w 29977421 10.14740/jocmr3503w PMC6031252

[2] Mete O, Erickson LA, Juhlin CC et al (2022) Overview of the 2022 WHO classification of adrenal cortical tumors. Endocr Pathol 33:155–196. 10.1007/s12022-022-09710-8 35288842 10.1007/s12022-022-09710-8 PMC8920443

[3] Else T, Kim AC, Sabolch A (2014) Adrenocortical carcinoma. Endocr Rev 35:282–326. 10.1210/er.2013-1029 24423978 10.1210/er.2013-1029 PMC3963263

[4] Fassnacht M, Libé R, Kroiss M, Allolio B (2011) Adrenocortical carcinoma: a clinician’s update. Nat Rev Endocrinol 7:323–335. 10.1038/nrendo.2010.235 21386792 10.1038/nrendo.2010.235

[5] Duregon E, Fassina A, Volante M et al (2013) The reticulin algorithm for adrenocortical tumor diagnosis: a multicentric validation study on 245 unpublished cases. Am J Surg Pathol 37:1433–1440. 10.1097/PAS.0b013e31828d387b 23774167 10.1097/PAS.0b013e31828d387b

[6] Wright JP, Montgomery KW, Tierney J et al (2018) Ectopic, retroperitoneal adrenocortical carcinoma in the setting of Lynch syndrome. Fam Cancer 17:381–385. 10.1007/s10689-017-0042-6 28940135 10.1007/s10689-017-0042-6 PMC10182439

[7] Cornejo KM, Afari HA, Sadow PM (2017) Adrenocortical carcinoma arising in an adrenal rest: a case report and review of the literature. Endocr Pathol 28:165–170. 10.1007/s12022-017-9472-9 28258518 10.1007/s12022-017-9472-9

[8] Yousaf A, Patterson J, Hobbs G et al (2020) Smoking is associated with adrenal adenomas and adrenocortical carcinomas: a nationwide multicenter analysis. Cancer Treatment and Research Communications 25:100206. 10.1016/j.ctarc.2020.100206 32871402 10.1016/j.ctarc.2020.100206

[9] Habra MA, Sukkari MA, Hasan A et al (2020) Epidemiological risk factors for adrenocortical carcinoma: a hospital-based case-control study. Int J Cancer 146:1836–1840. 10.1002/ijc.32534 31241762 10.1002/ijc.32534

[10] Else T, Rodriguez-Galindo C (2016) 5th international ACC symposium: hereditary predisposition to childhood ACC and the associated molecular phenotype: 5th International ACC Symposium Session: not just for kids! Horm Cancer 7:36–39. 10.1007/s12672-015-0244-z 26660147 10.1007/s12672-015-0244-z PMC10355924

[11] Juhlin CC, Bertherat J, Giordano TJ et al (2021) What did we learn from the molecular biology of adrenal cortical neoplasia? From histopathology to translational genomics. Endocr Pathol 32:102–133. 10.1007/s12022-021-09667-0 33534120 10.1007/s12022-021-09667-0

[12] Raymond VM, Everett JN, Furtado LV et al (2013) Adrenocortical carcinoma is a Lynch syndrome-associated cancer. J Clin Oncol 31:3012–3018. 10.1200/JCO.2012.48.0988 23752102 10.1200/JCO.2012.48.0988 PMC3739861

[13] Domènech M, Grau E, Solanes A et al (2021) Characteristics of adrenocortical carcinoma associated with Lynch syndrome. J Clin Endocrinol Metab 106:318–325. 10.1210/clinem/dgaa833 33185660 10.1210/clinem/dgaa833

[14] Bertherat J (2012) Adrenocortical cancer in Carney complex: a paradigm of endocrine tumor progression or an association of genetic predisposing factors? J Clin Endocrinol Metab 97:387–390. 10.1210/jc.2011-3327 22312093 10.1210/jc.2011-3327

[15] Shiroky JS, Lerner-Ellis JP, Govindarajan A et al (2018) Characteristics of adrenal masses in familial adenomatous polyposis. Dis Colon Rectum 61:679–685. 10.1097/DCR.0000000000001008 29377868 10.1097/DCR.0000000000001008

[16] Bertoin F, Letouzé E, Grignani P et al (2015) Genome-wide paternal uniparental disomy as a cause of Beckwith-Wiedemann syndrome associated with recurrent virilizing adrenocortical tumors. Horm Metab Res 47:497–503. 10.1055/s-0034-1394371 25365508 10.1055/s-0034-1394371

[17] Gatta-Cherifi B, Chabre O, Murat A et al (2012) Adrenal involvement in MEN1. Analysis of 715 cases from the Groupe d’étude des Tumeurs Endocrines database. Eur J Endocrinol 166:269–279. 10.1530/EJE-11-0679 22084155 10.1530/EJE-11-0679

[18] Menon RK, Ferrau F, Kurzawinski TR et al (2014) Adrenal cancer in neurofibromatosis type 1: case report and DNA analysis. Endocrinol Diabetes Metab Case Rep 2014:140074. 10.1530/EDM-14-0074 25520849 10.1530/EDM-14-0074 PMC4241507

[19] Else T, Lerario AM, Everett J et al (2017) Adrenocortical carcinoma and succinate dehydrogenase gene mutations: an observational case series. Eur J Endocrinol 177:439–444. 10.1530/EJE-17-0358 28819017 10.1530/EJE-17-0358

[20] Guo X, Chen H, Fu H, Wu H (2017) Hereditary leiomyomatosis and renal cell carcinoma syndrome combined with adrenocortical carcinoma on 18F-FDG PET/CT. Clin Nucl Med 42:692–694. 10.1097/RLU.0000000000001760 28737576 10.1097/RLU.0000000000001760

[21] Raygada M, Raffeld M, Bernstein A et al (2021) Case report of adrenocortical carcinoma associated with double germline mutations in *MSH2* and *RET*. Am J Med Genet A 185:1282–1287. 10.1002/ajmg.a.62099 33615670 10.1002/ajmg.a.62099 PMC7986073

[22] Fassnacht M, Dekkers O, Else T (2018) European Society of Endocrinology clinical practice guidelines on the management of adrenocortical carcinoma in adults, in collaboration with the European Network for the Study of Adrenal Tumors. Eur J Endocrinol 179:G1–G46. 10.1530/EJE-18-0608 30299884 10.1530/EJE-18-0608

[23] Kiseljak-Vassiliades K, Bancos I, Hamrahian A (2020) American association of clinical endocrinology disease state clinical review on the evaluation and management of adrenocortical carcinoma in an adult: a practical approach. Endocr Pract 26:1366–1383. 10.4158/DSCR-2020-0567 33875173 10.4158/DSCR-2020-0567 PMC8058447

[24] de Reyniès A, Assié G, Rickman DS et al (2009) Gene expression profiling reveals a new classification of adrenocortical tumors and identifies molecular predictors of malignancy and survival. J Clin Oncol 27:1108–1115. 10.1200/JCO.2008.18.5678 19139432 10.1200/JCO.2008.18.5678

[25] Assié G, Letouzé E, Fassnacht M et al (2014) Integrated genomic characterization of adrenocortical carcinoma. Nat Genet 46:607–612. 10.1038/ng.2953 24747642 10.1038/ng.2953

[26] Barreau O, Assié G, Wilmot-Roussel H et al (2013) Identification of a CpG island methylator phenotype in adrenocortical carcinomas. J Clin Endocrinol Metab 98:E174–184. 10.1210/jc.2012-2993 23093492 10.1210/jc.2012-2993

[27] Assié G, Jouinot A, Fassnacht M et al (2019) Value of molecular classification for prognostic assessment of adrenocortical carcinoma. JAMA Oncol 5:1440–1447. 10.1001/jamaoncol.2019.1558 31294750 10.1001/jamaoncol.2019.1558 PMC6624825

[28] Jouinot A, Lippert J, Sibony M et al (2022) Transcriptome in paraffin samples for the diagnosis and prognosis of adrenocortical carcinoma. Eur J Endocrinol 186:607–617. 10.1530/EJE-21-1228 35266879 10.1530/EJE-21-1228 PMC9066577

[29] Lippert J, Dischinger U, Appenzeller S et al (2023) Performance of DNA-based biomarkers for classification of adrenocortical carcinoma: a prognostic study. Eur J Endocrinol 189:262–270. 10.1093/ejendo/lvad112 37590967 10.1093/ejendo/lvad112

[30] Ayala-Ramirez M, Jasim S, Feng L et al (2013) Adrenocortical carcinoma: clinical outcomes and prognosis of 330 patients at a tertiary care center. Eur J Endocrinol 169:891–899. 10.1530/EJE-13-0519 24086089 10.1530/EJE-13-0519 PMC4441210

[31] Volante M, Bollito E, Sperone P et al (2009) Clinicopathological study of a series of 92 adrenocortical carcinomas: from a proposal of simplified diagnostic algorithm to prognostic stratification. Histopathology 55:535–543. 10.1111/j.1365-2559.2009.03423.x 19912359 10.1111/j.1365-2559.2009.03423.x

[32] Giordano TJ, Berney D, de Krijger RR (2021) Data set for reporting of carcinoma of the adrenal cortex: explanations and recommendations of the guidelines from the International Collaboration on Cancer Reporting. Hum Pathol 110:50–61. 10.1016/j.humpath.2020.10.001 33058949 10.1016/j.humpath.2020.10.001

[33] Weiss LM, Medeiros LJ, Vickery AL (1989) Pathologic features of prognostic significance in adrenocortical carcinoma. Am J Surg Pathol 13:202–206. 10.1097/00000478-198903000-00004 2919718 10.1097/00000478-198903000-00004

[34] Bisceglia M, Ludovico O, Di Mattia A et al (2004) Adrenocortical oncocytic tumors: report of 10 cases and review of the literature. Int J Surg Pathol 12:231–243. 10.1177/106689690401200304 15306935 10.1177/106689690401200304

[35] Aubert S, Wacrenier A, Leroy X et al (2002) Weiss system revisited: a clinicopathologic and immunohistochemical study of 49 adrenocortical tumors. Am J Surg Pathol 26:1612–1619. 10.1097/00000478-200212000-00009 12459628 10.1097/00000478-200212000-00009

[36] Libé R, Borget I, Ronchi CL et al (2015) Prognostic factors in stage III-IV adrenocortical carcinomas (ACC): an European Network for the Study of Adrenal Tumor (ENSAT) study. Ann Oncol 26:2119–2125. 10.1093/annonc/mdv329 26392430 10.1093/annonc/mdv329

[37] Mete O, Gucer H, Kefeli M, Asa SL (2018) Diagnostic and prognostic biomarkers of adrenal cortical carcinoma. Am J Surg Pathol 42:201–213. 10.1097/PAS.0000000000000943 28877067 10.1097/PAS.0000000000000943

[38] Fuhrman SA, Lasky LC, Limas C (1982) Prognostic significance of morphologic parameters in renal cell carcinoma. Am J Surg Pathol 6:655–663. 10.1097/00000478-198210000-00007 7180965 10.1097/00000478-198210000-00007

[39] Wieneke JA, Thompson LDR, Heffess CS (2003) Adrenal cortical neoplasms in the pediatric population: a clinicopathologic and immunophenotypic analysis of 83 patients. Am J Surg Pathol 27:867–881. 10.1097/00000478-200307000-00001 12826878 10.1097/00000478-200307000-00001

[40] Riedmeier M, Thompson LDR, Molina CAF et al (2023) Prognostic value of the Weiss and Wieneke (AFIP) scoring systems in pediatric ACC - a mini review. Endocr Relat Cancer 30:e220259. 10.1530/ERC-22-0259 36753311 10.1530/ERC-22-0259

[41] Ishikawa Y, Yamazaki Y, Tezuka Y et al (2024) Histopathological analysis of tumor microenvironment in adrenocortical carcinoma: possible effects of in situ disorganized glucocorticoid production on tumor immunity. J Steroid Biochem Mol Biol 238:106462. 10.1016/j.jsbmb.2024.106462 38232786 10.1016/j.jsbmb.2024.106462

[42] de Krijger RR, Papathomas TG (2012) Adrenocortical neoplasia: evolving concepts in tumorigenesis with an emphasis on adrenal cortical carcinoma variants. Virchows Arch 460:9–18. 10.1007/s00428-011-1166-y 22086150 10.1007/s00428-011-1166-y PMC3267029

[43] Duregon E, Volante M, Cappia S et al (2011) Oncocytic adrenocortical tumors: diagnostic algorithm and mitochondrial DNA profile in 27 cases. Am J Surg Pathol 35:1882–1893. 10.1097/PAS.0b013e31822da401 21989346 10.1097/PAS.0b013e31822da401

[44] Hsieh M-S, Chen J-H, Lin L-W (2011) Myxoid adrenal cortical carcinoma presenting as primary hyperaldosteronism: case report and review of the literature. Int J Surg Pathol 19:803–807. 10.1177/1066896909356925 20444728 10.1177/1066896909356925

[45] Papotti M, Volante M, Duregon E et al (2010) Adrenocortical tumors with myxoid features: a distinct morphologic and phenotypical variant exhibiting malignant behavior. Am J Surg Pathol 34:973–983. 10.1097/PAS.0b013e3181e2b726 20534995 10.1097/PAS.0b013e3181e2b726

[46] Branham Z, Fox AD, Ullah A et al (2022) Insights into clinical features and outcomes of adrenal cortical carcinosarcoma. Diagnostics 12:2419. 10.3390/diagnostics12102419 36292108 10.3390/diagnostics12102419 PMC9600293

[47] Papathomas TG, Duregon E, Korpershoek E et al (2016) Sarcomatoid adrenocortical carcinoma: a comprehensive pathological, immunohistochemical, and targeted next-generation sequencing analysis. Hum Pathol 58:113–122. 10.1016/j.humpath.2016.08.006 27589897 10.1016/j.humpath.2016.08.006

[48] Zheng S, Cherniack AD, Dewal N et al (2016) Comprehensive pan-genomic characterization of adrenocortical carcinoma. Cancer Cell 29:723–736. 10.1016/j.ccell.2016.04.002 27165744 10.1016/j.ccell.2016.04.002 PMC4864952

[49] Minner S, Schreiner J, Saeger W (2021) Adrenal cancer: relevance of different grading systems and subtypes. Clin Transl Oncol 23:1350–1357. 10.1007/s12094-020-02524-2 33818702 10.1007/s12094-020-02524-2 PMC8192347

[50] Hayashi T, Gucer H, Mete O (2014) A mimic of sarcomatoid adrenal cortical carcinoma: epithelioid angiosarcoma occurring in adrenal cortical adenoma. Endocr Pathol 25:404–409. 10.1007/s12022-014-9330-y 25246132 10.1007/s12022-014-9330-y

[51] Weiss LM (1984) Comparative histologic study of 43 metastasizing and nonmetastasizing adrenocortical tumors. Am J Surg Pathol 8:163–169. 10.1097/00000478-198403000-00001 6703192 10.1097/00000478-198403000-00001

[52] Pennanen M, Heiskanen I, Sane T et al (2015) Helsinki score-a novel model for prediction of metastases in adrenocortical carcinomas. Hum Pathol 46:404–410. 10.1016/j.humpath.2014.11.015 25582500 10.1016/j.humpath.2014.11.015

[53] Tissier F, Aubert S, Leteurtre E (2012) Adrenocortical tumors: improving the practice of the Weiss system through virtual microscopy: a National Program of the French Network INCa-COMETE. Am J Surg Pathol 36:1194–1201. 10.1097/PAS.0b013e31825a6308 22790860 10.1097/PAS.0b013e31825a6308

[54] Duregon E, Cappellesso R, Maffeis V et al (2017) Validation of the prognostic role of the “Helsinki score” in 225 cases of adrenocortical carcinoma. Hum Pathol 62:1–7. 10.1016/j.humpath.2016.09.035 27916625 10.1016/j.humpath.2016.09.035

[55] Renaudin K, Smati S, Wargny M et al (2018) Clinicopathological description of 43 oncocytic adrenocortical tumors: importance of Ki-67 in histoprognostic evaluation. Mod Pathol 31:1708–1716. 10.1038/s41379-018-0077-8 29921900 10.1038/s41379-018-0077-8

[56] Jangir H, Ahuja I, Agarwal S et al (2023) Pediatric adrenocortical neoplasms: a study comparing three histopathological scoring systems. Endocr Pathol 34:213–223. 10.1007/s12022-023-09767-z 37160532 10.1007/s12022-023-09767-z

[57] Angelousi A, Kyriakopoulos G, Athanasouli F et al (2021) The role of immunohistochemical markers for the diagnosis and prognosis of adrenocortical neoplasms. J Pers Med 11:208. 10.3390/jpm11030208 33804047 10.3390/jpm11030208 PMC8001501

[58] Dalino Ciaramella P, Vertemati M, Petrella D et al (2017) Analysis of histological and immunohistochemical patterns of benign and malignant adrenocortical tumors by computerized morphometry. Pathol Res Pract 213:815–823. 10.1016/j.prp.2017.03.004 28554744 10.1016/j.prp.2017.03.004

[59] Martins-Filho SN, Almeida MQ, Soares I et al (2021) Clinical impact of pathological features including the Ki-67 labeling index on diagnosis and prognosis of adult and pediatric adrenocortical tumors. Endocr Pathol 32:288–300. 10.1007/s12022-020-09654-x 33443677 10.1007/s12022-020-09654-x

[60] Giordano TJ (2011) The argument for mitotic rate-based grading for the prognostication of adrenocortical carcinoma. Am J Surg Pathol 35:471–473. 10.1097/PAS.0b013e31820bcf21 21383612 10.1097/PAS.0b013e31820bcf21

[61] Duregon E, Molinaro L, Volante M et al (2014) Comparative diagnostic and prognostic performances of the hematoxylin-eosin and phospho-histone H3 mitotic count and Ki-67 index in adrenocortical carcinoma. Mod Pathol 27:1246–1254. 10.1038/modpathol.2013.230 24434900 10.1038/modpathol.2013.230

[62] Mete O, Asa SL, Giordano TJ et al (2018) Immunohistochemical biomarkers of adrenal cortical neoplasms. Endocr Pathol 29:137–149. 10.1007/s12022-018-9525-8 29542002 10.1007/s12022-018-9525-8

[63] Campbell AN, Choi WJ, Chi ES et al (2024) Steroidogenic factor-1 form and function: from phospholipids to physiology. Adv Biol Regul 91:100991. 10.1016/j.jbior.2023.100991 37802761 10.1016/j.jbior.2023.100991 PMC10922105

[64] Duregon E, Volante M, Giorcelli J et al (2013) Diagnostic and prognostic role of steroidogenic factor 1 in adrenocortical carcinoma: a validation study focusing on clinical and pathologic correlates. Hum Pathol 44:822–828. 10.1016/j.humpath.2012.07.025 23158211 10.1016/j.humpath.2012.07.025

[65] Sbiera S, Schmull S, Assie G et al (2010) High diagnostic and prognostic value of steroidogenic factor-1 expression in adrenal tumors. J Clin Endocrinol Metab 95:E161–171. 10.1210/jc.2010-0653 20660055 10.1210/jc.2010-0653

[66] González-Sáez L, Pita-Fernández S, Lorenzo-Patiño MJ et al (2011) Primary melanoma of the adrenal gland: a case report and review of the literature. J Med Case Rep 5:273. 10.1186/1752-1947-5-273 21722390 10.1186/1752-1947-5-273 PMC3141716

[67] Bennett JA, Braga AC, Pinto A et al (2018) Uterine PEComas: a morphologic, immunohistochemical, and molecular analysis of 32 tumors. Am J Surg Pathol 42:1370–1383. 10.1097/PAS.0000000000001119 30001237 10.1097/PAS.0000000000001119 PMC6133752

[68] Wang A-X, Tian T, Liu L-B et al (2023) TFEB rearranged renal cell carcinoma: pathological and molecular characterization of 10 cases, with novel clinical implications: a single center 10-year experience. Biomedicines 11:245. 10.3390/biomedicines11020245 36830782 10.3390/biomedicines11020245 PMC9952947

[69] Mete O, Pakbaz S, Lerario AM et al (2021) Significance of alpha-inhibin expression in pheochromocytomas and paragangliomas. Am J Surg Pathol 45:1264–1273. 10.1097/PAS.0000000000001715 33826547 10.1097/PAS.0000000000001715

[70] Margonis GA, Kim Y, Prescott JD et al (2016) Adrenocortical carcinoma: impact of surgical margin status on long-term outcomes. Ann Surg Oncol 23:134–141. 10.1245/s10434-015-4803-x 26286195 10.1245/s10434-015-4803-x PMC4955567

[71] Bilimoria KY, Shen WT, Elaraj D et al (2008) Adrenocortical carcinoma in the United States: treatment utilization and prognostic factors. Cancer 113:3130–3136. 10.1002/cncr.23886 18973179 10.1002/cncr.23886

[72] Luconi M, Cantini G, van Leeuwaarde RS et al (2023) Prognostic value of microscopic tumor necrosis in adrenal cortical carcinoma. Endocr Pathol 34:224–233. 10.1007/s12022-023-09760-6 36952130 10.1007/s12022-023-09760-6 PMC10264263

[73] Beuschlein F, Weigel J, Saeger W et al (2015) Major prognostic role of Ki67 in localized adrenocortical carcinoma after complete resection. J Clin Endocrinol Metab 100:841–849. 10.1210/jc.2014-3182 25559399 10.1210/jc.2014-3182

[74] Yamazaki Y, Nakamura Y, Shibahara Y (2016) Comparison of the methods for measuring the Ki-67 labeling index in adrenocortical carcinoma: manual versus digital image analysis. Hum Pathol 53:41–50. 10.1016/j.humpath.2015.10.017 26980031 10.1016/j.humpath.2015.10.017

[75] Papathomas TG, Pucci E, Giordano TJ (2016) An international Ki67 reproducibility study in adrenal cortical carcinoma. Am J Surg Pathol 40:569–576. 10.1097/PAS.0000000000000574 26685085 10.1097/PAS.0000000000000574

[76] Brondani VB, Lacombe AMF, Mariani BM de P, et al (2021) Low protein expression of both ATRX and ZNRF3 as novel negative prognostic markers of adult adrenocortical carcinoma. Int J Mol Sci 22:1238. 10.3390/ijms22031238 10.3390/ijms22031238 PMC7866180 33513905

[77] Araujo-Castro M, Pascual-Corrales E, Molina-Cerrillo J, Alonso-Gordoa T (2021) Immunotherapy in adrenocortical carcinoma: predictors of response, efficacy, safety, and mechanisms of resistance. Biomedicines 9:304. 10.3390/biomedicines9030304 33809752 10.3390/biomedicines9030304 PMC8002272

[78] Gomez-Sanchez CE, Gomez-Sanchez EP, Nishimoto K (2020) Immunohistochemistry of the human adrenal CYP11B2 in normal individuals and in patients with primary aldosteronism. Horm Metab Res 52:421–426. 10.1055/a-1139-2079 32289837 10.1055/a-1139-2079 PMC7299743

[79] Germano A, Saba L, De Francia S et al (2018) CYP11B1 has no role in mitotane action and metabolism in adrenocortical carcinoma cells. PLoS ONE 13:e0196931. 10.1371/journal.pone.0196931 29734384 10.1371/journal.pone.0196931 PMC5937768

[80] Yan X, Guo Z-X, Yu D-H et al (2021) Identification and validation of a novel prognosis prediction model in adrenocortical carcinoma by integrative bioinformatics analysis, statistics, and machine learning. Front Cell Dev Biol 9:671359. 10.3389/fcell.2021.671359 34164395 10.3389/fcell.2021.671359 PMC8215582

[81] Gupta S, Won H, Chadalavada K et al (2022) TERT copy number alterations, promoter mutations and rearrangements in adrenocortical carcinomas. Endocr Pathol 33:304–314. 10.1007/s12022-021-09691-0 34549366 10.1007/s12022-021-09691-0 PMC9135779

[82] Soon PSH, Tacon LJ, Gill AJ et al (2009) Mir-195 and miR-483-5p identified as predictors of poor prognosis in adrenocortical cancer. Clin Cancer Res 15:7684–7692. 10.1158/1078-0432.CCR-09-1587 19996210 10.1158/1078-0432.CCR-09-1587

[83] Mohan DR, Lerario AM, Else T et al (2019) Targeted assessment of G0S2 methylation identifies a rapidly recurrent, routinely fatal molecular subtype of adrenocortical carcinoma. Clin Cancer Res 25:3276–3288. 10.1158/1078-0432.CCR-18-2693 30770352 10.1158/1078-0432.CCR-18-2693 PMC7117545

[84] Ronchi CL, Sbiera S, Volante M et al (2014) CYP2W1 is highly expressed in adrenal glands and is positively associated with the response to mitotane in adrenocortical carcinoma. PLoS ONE 9:e105855. 10.1371/journal.pone.0105855 25144458 10.1371/journal.pone.0105855 PMC4140842

[85] Takaishi T, Kokubo Y, Kondo K et al (2025) Automated detection and classification of adrenal masses on CT using two annotation methods: segmentation vs. bounding box. European Journal of Radiology Artificial Intelligence 1:100003. 10.1016/j.ejrai.2025.100003

[86] Zhang J, Chao Y, Yao K, Guo S (2024) Differentiating between adrenocortical carcinoma and pheochromocytoma by radiomics features at CT: a multi-institutional retrospective study. JCO 42:4603–4603. 10.1200/JCO.2024.42.16_suppl.4603

[87] Tucci L, Vara G, Morelli V et al (2024) Prediction of adrenal masses nature through texture analysis and deep learning: preliminary results from ENS@T RADIO-AI multicentric study. EJEA. 10.1530/endoabs.99.OC11.3

[88] Kong J, Luo M, Huang Y et al (2025) More than meets the eye: predicting adrenocortical carcinoma outcomes with pathomics. Eur J Endocrinol 192:61–72. 10.1093/ejendo/lvae162 39871591 10.1093/ejendo/lvae162

